# Mendeliome sequencing enables differential diagnosis and treatment of neonatal lactic acidosis

**DOI:** 10.1186/s40348-016-0050-x

**Published:** 2016-06-17

**Authors:** Walid Fazeli, Mert Karakaya, Peter Herkenrath, Anne Vierzig, Jörg Dötsch, Jürgen-Christoph von Kleist-Retzow, Sebahattin Cirak

**Affiliations:** Department of Pediatrics, University Hospital Cologne, Kerpener Str. 62, 50937 Cologne, Germany; Institute of Human Genetics, University Hospital Cologne, Cologne, Germany; Center for Molecular Medicine, University of Cologne, Cologne, Germany

**Keywords:** Mendeliome sequencing, *PDH* deficiency, Ketogenic diet, *PDHX*

## Abstract

**Background:**

Neonatal lactic acidosis can be associated to severe inborn errors of metabolism. Rapid identification of the underlying disorder may improve the clinical management through reliable counseling of the parents and adaptation of the treatment.

**Methods:**

We present the case of a term newborn with persistent hypoglycemia on postnatal day 1, who developed severe lactic acidosis, aggravating under intravenous glucose administration. Routine metabolic investigations revealed elevated pyruvate and lactate levels in urine, and magnetic resonance spectroscopy showed a lactic acid peak and decreased N-acetylaspartate levels. Mitochondrial disorders, e.g., pyruvate dehydrogenase (PDH) deficiency, were the major differential diagnoses. However, both hypoglycemia and the elevated lactate to pyruvate ratio in serum (=55.2) were not typical for PDH deficiency. We used “Mendeliome sequencing”, a next-generation sequencing approach targeting all genes which have been previously linked to single-gene disorders, to obtain the correct diagnosis.

**Results:**

On day 27 of life, we identified a homozygous stop mutation in the *PDHX* gene, causing pyruvate dehydrogenase E3-binding protein deficiency. After starting the ketogenic diet, the infant recovered and is showing delayed but progressive development.

**Conclusions:**

Mendeliome sequencing was successfully used to disentangle the underlying cause of severe neonatal lactic acidosis. Indeed, it is one of several targeted sequencing approaches that allow rapid and reliable counseling of the parents, adaptation of the clinical management, and renunciation of unnecessary, potentially invasive and often costly diagnostic measures.

**Electronic supplementary material:**

The online version of this article (doi:10.1186/s40348-016-0050-x) contains supplementary material, which is available to authorized users.

## Background

Neonatal lactic acidosis can be associated to a heterogeneous spectrum of causes, ranging from benign (e.g., protracted postnatal adaptation) to severe, potentially life-threatening conditions, e.g., disorders of energy metabolism such as deficiency of the pyruvate dehydrogenase (PDH) complex. PDH deficiency is the most common biochemically proven cause of congenital lactic acidosis [1] and thus an important differential diagnosis, particularly as treatment approaches exist. The PDH complex is a large (approx. 8 MDa) nuclear-encoded multi-enzyme complex located in the mitochondrial matrix and formed by three functional subunits (E1, E2, and E3 plus the E3-binding protein (E3BP)). It catalyzes the oxidation of pyruvate to acetyl coenzyme A and thereby links cytoplasmic glycolysis to the intra-mitochondrial tricarboxylic acid cycle. Deficiency of the PDH complex leads to accumulation of pyruvate and lactic acid as well as impaired mitochondrial energy restoration which is particularly harmful to the brain leading to impaired life expectancy and neurocognitive development. PDH deficiency can be caused by mutations in several different genes (e.g., *PDHA1* [OMIM 300502], *PDHX* [608769], *PDHB* [179060], *DLAT* [608770]; see [2] for overview). Additionally, an increasing number of mutations have been found in genes that encode for co-factors of pyruvate oxidation (e.g., thiamine), resembling the wide clinical spectrum of PDH deficiency [2] that embraces phenotypes which are primarily metabolic (i.e., severe neonatal lactic acidosis) and those which are rather neurologic (i.e., muscular hypotonia and congenital brain malformations, e.g., agenesis of corpus callosum). The E3BP of the PDH complex is encoded by the pyruvate dehydrogenase complex-component X *(PDHX)* gene. After *PDHA1*, *PDHX* is the second most commonly mutated gene in PDH deficiency [3]. So far, a genotype-phenotype correlation has not been established due to the different affected domains of E3BP and the heterogeneity of the clinical features [4].

In contrast to other possible causes of neonatal lactic acidosis, there is currently no fully satisfying treatment available for patients with PDH deficiency [5]. However, ketogenic diet (KD) has been shown to positively influence the outcome of patients with PDH deficiency as it provides an alternative energy source and thereby bypasses the detrimental energy deprivation [2, 6]. In case PDH deficiency underlies neonatal lactic acidosis, early initiation of KD supposedly correlates with improved long-term outcome [7]. Precise diagnosis before the start of KD is required as there are contra-indications in some cases of lactic acidosis; KD should be avoided in some defects of the mitochondrial respiratory chain [8, 9] and in pyruvate carboxylase deficiency [8, 10] as it can enhance lactic acidosis and deteriorate the clinical situation [9, 10]. Currently, the diagnostic approach to monogenetic disorders such as PDH deficiency is experiencing pivotal changes. While previously, newborns and infants often underwent invasive, time-consuming, and costly diagnostic procedures—yet in some cases with ambiguous and inconclusive results [11]—next-generation sequencing approaches are increasingly implemented in daily clinical practice. Here, we illustrate in a case of neonatal lactic acidosis that a disease gene panel based on a targeted next-generation sequencing (NGS) method—an approach that we term “Mendeliome sequencing”—can be successfully applied. This NGS approach allows targeted sequencing of all disease genes listed in the Online Mendelian Inheritance of Man (OMIM) database.

## Methods

The study was approved by the institutional review board of the Ethics Committee of the University Hospital of Cologne. Informed consent for research and publication was obtained from the family.

### Case description

We present the case of a male newborn of Turkish-origin parents who was born at term at a peripheral children’s hospital. The parents stated to be non-consanguineous but belonged both to an endogamic subpopulation from Turkey. The patient’s younger sister was healthy while the older sister suffered from agenesis of corpus callosum and severe psychomotor retardation; as the parents have been rejecting any genetic counseling, the etiology of her disease remains unknown. After a complicated pregnancy (maternal diabetes mellitus, placenta previa, HELLP syndrome), the boy was born by secondary caesarian section due to placental abruption. Initial postnatal adaptation was unremarkable, but routine check of blood sugar levels (in response to maternal diabetes mellitus) first revealed repetitive neonatal hypoglycemia (8 h of age) and later on spontaneous development of moderate lactic acidosis (13 h of age: lactic acid 8.6 mmol/l (normal, ≤ 2.1 mmol/l); pH 7336). As hypoglycemia did not improve under repetitive feeding, intravenous (i.v.) glucose administration (6 mg/kg body weight/min.) was necessary (starting at 14 h of age). However, after a bolus application of glucose (10 %, 2 ml/kg body weight), the patient developed hyperglycemia (max. 397 mg/dl) and further worsening of lactic acidosis (24 h of age: lactic acid 16 mmol/l; pH 7037) which barely improved despite subsequent reduction of glucose supply and buffering with bicarbonate. Thus, an inborn error of metabolism was suspected, and the boy was transferred to the University Children’s Hospital 25 h after birth. Though he remained clinically unimpaired, lactic acidosis worsened (27 h of age: pH 7.0, lactic acid 18 mmol/l, base excess −21). Further investigations showed ketone bodies in urine as well as mildly elevated liver enzyme and CRP levels, in response to which antibiotics were started (and again terminated 5 days later after exclusion of neonatal infection; cultures of blood and cerebrospinal fluid were unremarkable). Despite extensive metabolic screening, the diagnosis remained elusive (normal values for newborn screening, ammonium, serum acylcarnitines/carnitine, urine amino acids, urine organic acids, very long chain fatty acids (VLCFA), homocystein, purines/pyrimidines in urine; plasma amino acids: elevated alanine secondary to lactic acidosis [1054 μmol/l, normal <710 μmol/l]). A mitochondrial disorder was now suggested as the underlying cause for several reasons: aggravation of lactic acidosis under i.v. glucose supply; increased levels of pyruvate and lactic acid in urine (both >5000 mmol/mol creatinine, normal: lactic acid <285, pyruvate <130); and finally, the findings of a magnetic resonance (MR) spectroscopy performed on day 6 after birth, that showed lactic acid peaks in the right occipital and frontal cortical areas under involvement of the subcortical white matter. Alterations of brain morphology, in particular agenesis of corpus callosum as seen in his older sister and as previously described for PDH deficiency, were not present. Buffering via bicarbonate administration was stopped on postnatal day 3, and tentative treatment with thiamine (75 mg/kg body weight/day) and carnitine (100 mg/kg body weight/day) was started. However, two findings—the initially observed refractory hypoglycemia and the elevated lactate to pyruvate ratio in the blood (55.2 = 6.9 mmol/l [lactate] to 0.125 mmol/l [pyruvate] in blood samples taken simultaneously)—were not characteristic of PDH deficiency and could have been each of them caused by many other inborn errors of metabolism [2, 4]. On postnatal day 16, we therefore applied a genetic approach to identify the underlying disorder.

### Sample preparation and sequencing analysis

DNA was extracted from blood with standard protocols. We performed next-generation sequencing for Mendelian diseases—which we term Mendeliome sequencing—in order to clarify the diagnosis. Fifty nanogram of genomic DNA (gDNA) were used for the TruSight One Sequencing Panel (Illumina) library prep protocol (Number 15046433, 2013). After quality control and quantification, the library was pooled and enriched for 4813 genes associated to monogenetic disorders. The pool underwent qPCR as a final control step and was loaded on an Illumina MiSeq benchtop sequencer using v3 chemistry and a 2 × 150 bp read length. The Cologne Center for Genomics VARBANK pipeline v.2.12 (https://varbank.ccg.uni-koeln.de/) was used for data analysis (Table 1).

**Table 1:** Pipeline and filter parameters used for Mendeliome sequencing (filter metrics)

Filter parameter	Patient
Reads variation allele frequency range	75–100 %
Maximal number of allowed variations per gene	10
Maximal number seen in epilepsy in-house DM (*n* = 511)	5
Maximal number seen in structural in-house DM (*n* = 611)	10
Maximal population variation frequency	0.1 %
Minimal read coverage	6
Minimal variation quality	10
Maximal target distance	100
Transcryptic biotypes	protein_coding
Variation locations	COMPOSED (variation spans different parts of a gene), intron, CDS (coding sequence)
Consequence types	Protein structure affected; cryptic 5′SS/3′SS activation; strong 5′SS/3′SS effects

## Results

### Genetic work-up

By sequencing, a mean coverage of 82 % was reached, 10× coverage was 97.5 % for target sequences and 30× coverage was 87.1 % on target (Additional file 1: Table S1). After analysis by our in-house software VARBANK v.2.12 (https://varbank.ccg.uni-koeln.de/), we deciphered the underlying condition within 11 days, i.e., at postnatal day 27. Initially, we looked for homozygous variants located in the shared runs of homozygosity (ROH). For completion, we also performed a further analysis of other possible heterozygous variants which did not reveal any mutation that could explain the biochemical and clinical features in this newborn. After filters were applied, the number of variants was reduced to five. Further filtering was performed on the basis of quality of reads, frequency in the population, prediction of pathogenicity, and evolutionary conservation, leading to one disease-causing genetic variant (Table 2).

**Table 2:** Sequentially listed filtering steps for homozygous analysis

ROH	
Rare functional variants (RFV)	5
Non-synonymous coding, indels	4
RFVs in good sequence quality (*Q* > 100)	4
RFVs not reported in dbSNP database	2
Non-reported RFVs predicted to be disease causing	1

*ROH* regions of homozygosity

**Fig. 1:**
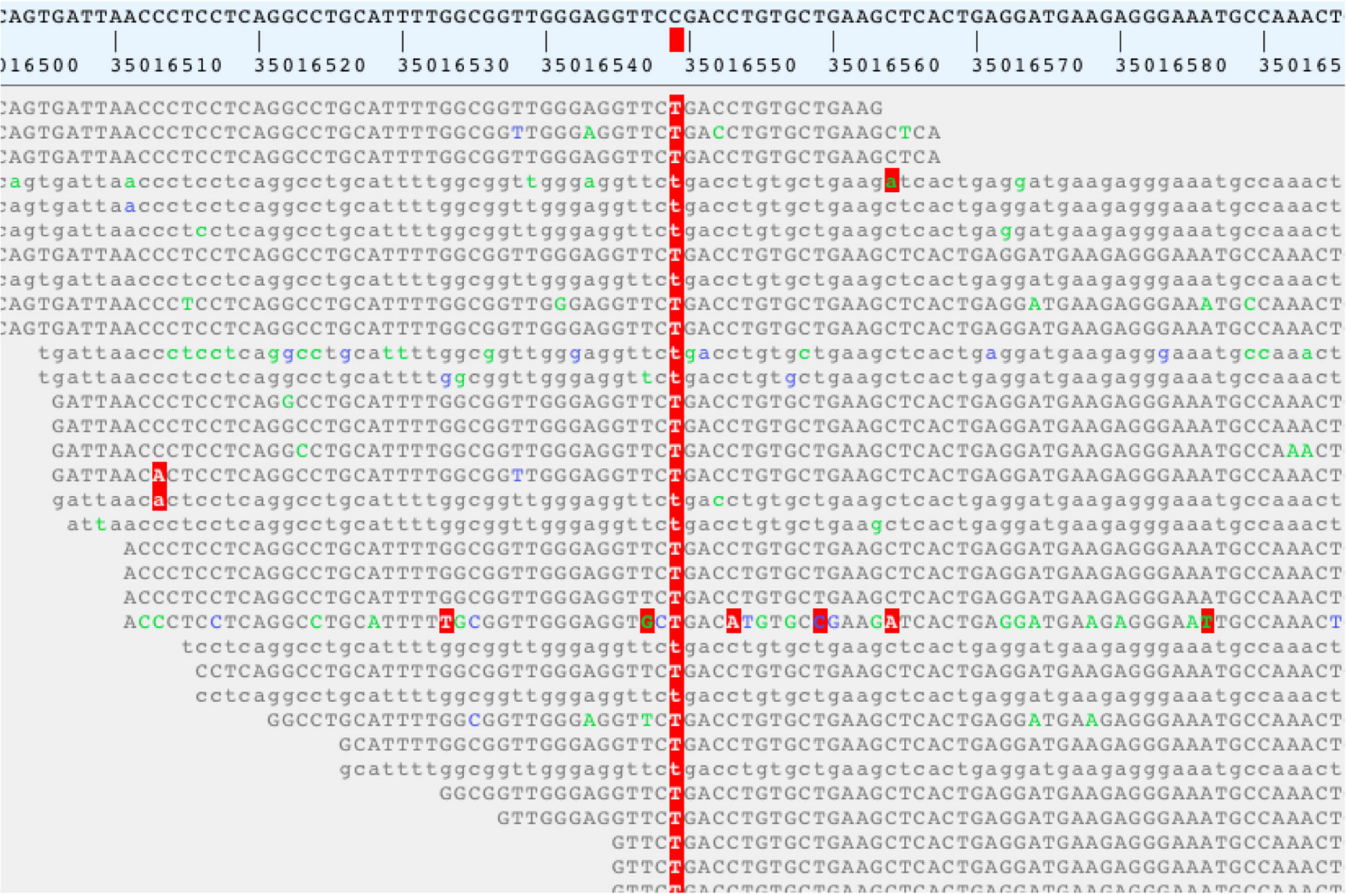
Mendeliome reads show *PDHX* mutation. Reads from the patient’s genome show homozygous change from C to T (https://varbank.ccg.uni-koeln.de/). *Above in black capital letters* are the genomic reference sequence and the hg19 coordinates. *Below* is the next-generation sequencing read alignment, *small and capital letters* correspond to different sequencing directions. The mutation is labeled in *red*

We diagnosed pyruvate dehydrogenase E3-binding protein deficiency (OMIM 245349) by detecting a homozygous stop mutation in the *PDHX* gene (c.1336C>T in exon 11 causing p.R446* according to NM_003477.2) (Fig. 1). The *PDHX* variant was tested by Sanger sequencing, and the mutation was shown to be co-segregating within the family. The p.R446* allele was not present in the Exome Aggregation Consortium (http://exac.broadinstitute.org/) and the Exome Variant Server (http://evs.gs.washington.edu/EVS/). We show the excellent coverage of the Mendeliome sequencing for the *PDHX* gene in Additional file 2: Figure S1.

## Discussion

We present a newborn with severe neonatal lactic acidosis in which we applied a NGS technique to identify pyruvate dehydrogenase E3-binding protein deficiency as the underlying disorder. Once the genetic diagnosis was made, KD (3:1) was started (on postnatal day 28) and lactic acid values were thereby normalized. Despite sufficient caloric intake (~180 kcal/kg body weight/day), the patient developed failure to thrive at the beginning of KD. Showing signs of exocrine pancreatic insufficiency (pancreatic elastase in stool, 72 μg/g stool; normal, >200), the boy received pancreatin as exogenous replacement therapy (for approximately 2 months). He profited of this treatment and showed adequate weight gain, even after termination of pancreatin treatment. In contrast to other rare forms of mitochondrial disorders, particularly Pearson syndrome [12], exocrine pancreas insufficiency has not been described in PDH deficiency. As discussed in a previous case report, this might represent a particular complication of KD treatment in very young infants [13]. At 7 months of age, our patient had developed a secondary microcephaly and had a moderate delay of motor development. Other comorbidities have not (yet) occurred.

The mutation (p.Arg446*) was recently described as a founder mutation in 60 % of the patients of the Roma population who presented with congenital lactic acidosis [14]. Later in life, these patients mostly suffer from spastic diplegia, epileptic seizures, cortical brain atrophy, ventricular enlargement, and mental retardation [14]. Though a genotype-phenotype correlation has not been well established for PDH deficiency, these findings suggest that this particular mutation could be associated to a severe outcome, underlining the need to counteract long-term developmental impairment as much as possible. Numerous case reports [7, 15] and a study in zebrafish [16] have shown the potential therapeutic benefit of KD in PDH deficiency. It has been suggested that early initiation of KD in PDH deficiency might improve patients’ long-term outcome [7]. This would also have socio-economic implications.

However, rapid diagnosis of PDH deficiency is challenging as it is often not possible to separate it from other mitochondrial disorders by mere clinical observation and specific metabolites/biochemical findings [2]. Indeed, we were misled by the lactate/pyruvate ratio in the blood (=55.2) that was unusually high for PDH deficiency [2, 4] but could have fitted to defects of the mitochondrial respiratory chain complex I or IV [17]. This indicates that caution needs to be warranted in the interpretation of the L/P ratio, especially if the ratio was only measured once, as in our case. Previously, the diagnosis of PDH deficiency was primarily based on laborious biochemical enzymatic assays of, e.g., muscle tissue or cultured fibroblasts usually being both time-consuming and potentially inconclusive, in particular in very young infants under 3 months of age. Furthermore, depending on the X-inactivation pattern, cases of X-chromosomal *PDHA1* deficiency can be missed with biochemical assays [2]. Thus, there is a need for fast and reliable methods to identify patients with PDH deficiency. Targeted next-generation sequencing panels have been shown to ease this need. Targeted gene sequencing panels may cover all (coding) exons of the human genome, i.e., whole-exome sequencing (WES), or a selection of exons in a limited number of relevant genes. Whole-genome sequencing (WGS) aims to cover the entire human genome including the non-coding regions. Recently, it has been shown that in child neurology practice, a substantial part of the patients profited from WGS because it led to the genetic identification of the underlying disorder [18]. The resulting implications are more than just therapeutic ones: the general clinical management of the patient and its family—e.g., by facilitating parents’ decision-making in end-of-life situations—may be substantially improved by fast genetic diagnoses. Targeted next-generation sequencing panels—such as for metabolic disorders, mitochondrial disorders or as in our case the Mendeliome—have several advantages. Compared to WGS or WES, a restriction to candidate genes—selected based on the clinical presentation of the patient and/or restricted to all known OMIM-listed genes—is cheaper and quicker and detects clinically relevant variant interpretation of known disease genes easier. From a broad perspective, sequencing off all OMIM-listed genes by the Mendeliome additionally offers the advantage to detect unusual links of clinical presentations and genetic mutations (genotype-phenotype correlations). Current studies of rapid WGS in neonatal settings show a high detection rate of deep intronic variants, which carries the problem of higher false positive rates and expensive, compelling, labor-intensive variant testing [19]. Targeted NGS panels (e.g., restricted on inborn errors of metabolism or the Mendeliome) have shorter turn-around times compared to standard WGS and WES. Rapid WGS however is comparably quick, providing the molecular diagnosis within 26 to 50 h [20–22]. However, this WGS approach needs expensive hardware and computational clusters that are only available in large genome centers of developed countries. Mendeliome sequencing has restrictions, of course. It has not been designed to detect deep intronic mutations, which currently can only be detected by WGS. Also, Mendeliome sequencing requires regular updates of the targeted genes. Further, low coverage in certain exons of a number of genes might lead to false negative results, requiring further technical optimization. However, our recent unpublished data shows that certain genomic regions are even elusive to WGS and can only be sequenced by special techniques (data not shown). Structural variants including copy number variants are difficult to detect with targeted gene panels using NGS. Although several algorithms based on coverage statistics have been developed, false positive and negative rates are very high in particular for heterozygous copy number variants. Further bioinformatic analysis with computational tools under development might make the identification of large structural variants and copy number variations more reliable in the future. In contrast, Mendeliome sequencing and other targeted NGS panels could be established in any molecular genetic laboratory and can be run by benchtop NGS machines while the data analysis can be performed on up-to-date standard computers.

The presented case shows that Mendeliome sequencing can be applied in case of neonatal lactic acidosis, providing the diagnosis and thereby enabling initiation of treatment. It took us 11 days from initiation of genetic investigation (on postnatal day 16) until finding of the diagnosis (on postnatal day 27). That was due to the fact that Mendeliome sequencing is not yet established in the daily clinical routine and that it is not a first-line diagnostic method. Thus, it was conducted in our research laboratory which obviously takes much longer. However, if this novel method is established in the clinical routine, Mendeliome sequencing is feasible within 72 h (Illumina Truesight One Manual 15046433, 2013). For cases of PDH deficiency in particular, Mendeliome sequencing is quicker than the previously used enzymatic assays. Our case of pyruvate dehydrogenase E3-binding protein deficiency could have equally been solved by targeted gene panels restricted on inborn errors of metabolism and/or mitochondrial disorders. The decision whether to choose smaller gene panels or the Mendeliome panel should be based on careful clinical judgment, which might also depend on the availability of subspecialty experts. Mendeliome sequencing is a novel diagnostic tool that might in the future become a valuable supplement to classical biochemical approaches. It is conceivable that Mendeliome could be preferentially applied in clinical settings with largely unspecific and undefined phenotypes and/or misleading biochemical findings (which we personally experienced in this particular case). The more specific a phenotype is (and the less ambiguous potential biochemical findings are), the more likely more restrictive targeted gene panels (e.g., for mitochondrial disorders) could potentially be used.

## Conclusions

In summary, this case shows the feasibility of Mendeliome sequencing as a method that can be useful in patients with neonatal lactic acidosis to rapidly identify cases of pyruvate dehydrogenase deficiency. Thus, together with numerous previous NGS publications, our case indicates that rapid genetic diagnosis via targeted sequencing panels (e.g., Mendeliome sequencing) may improve the long-term outcome of patients with treatable metabolic or other inherited disorders. The utility of next-generation sequencing panels in comparison to conventional metabolic testing needs to be validated in the clinical routine settings.

## Additional files

Additional file 1: Table S1. Total number of reads and coverage analysis of Mendeliome data. Additional file 2: Figure S1. Coverage analysis of the *PDHX* gene.

## Abbreviations

KD: ketogenic diet
NGS: next-generation sequencing
PDH: pyruvate dehydrogenase
WES: whole-exome sequencing
WGS: whole-genome sequencing
