# Supplementary material for: Mendeliome sequencing enables differential diagnosis and treatment of neonatal lactic acidosis
Source: Mol Cell Pediatr. 2016 Jun 17;3:22. doi: 10.1186/s40348-016-0050-x (PMC4912540; doi:10.1186/s40348-016-0050-x)
Supplement: Additional file 1: Table S1. — Total number of reads and coverage analysis of Mendeliome data. [file 40348_2016_50_MOESM1_ESM.pdf]

Table S1: Total number of reads and coverage analysis of exome data

	Coverage 2x	Coverage 10x	Coverage 20x	Coverage 30x	Mean Coverage	Total Reads	Unique Reads	Unique Mapped Reads
Patient	98.9	97.5	93.8	87.1	82	15282138	13017968	12417817
