# Supplementary material for: Mendeliome sequencing enables differential diagnosis and treatment of neonatal lactic acidosis
Source: Mol Cell Pediatr. 2016 Jun 17;3:22. doi: 10.1186/s40348-016-0050-x (PMC4912540; doi:10.1186/s40348-016-0050-x)
Supplement: Additional file 2: Figure S1. — Coverage analysis of the PDHX gene. [file 40348_2016_50_MOESM2_ESM.pdf]

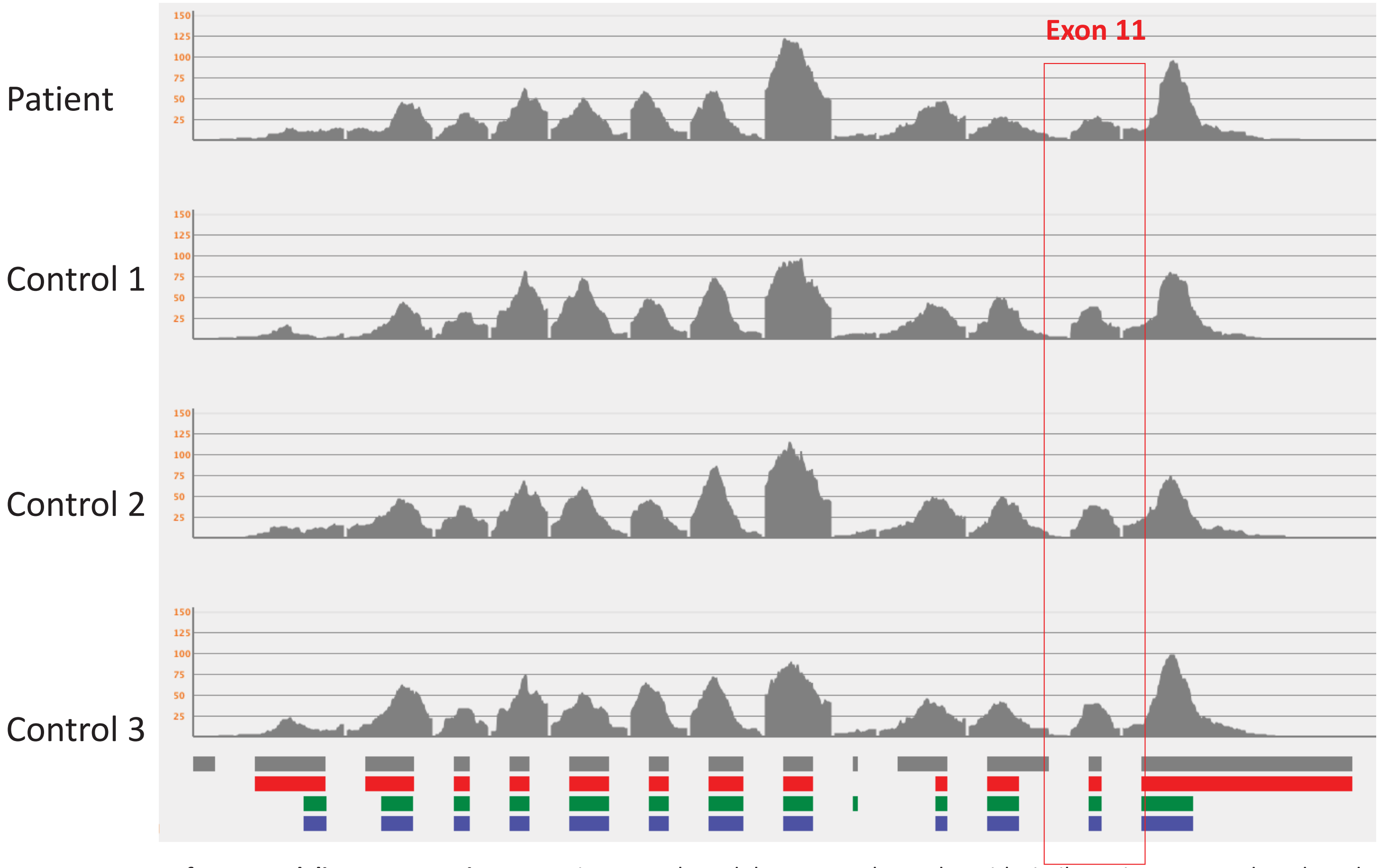

Figure S1: Coverage of the PDHX gene exons from Mendeliome sequencing. Patient sample and three control samples with similar unique mapped reads and mean coverage (1,236,964,975 and 81, respectively). The coverage of exon 11 in patient's sample with the truncating R446* mutation (http://varbank.ccg.uni-koeln.de) is marked. Patient's sample and three control samples with similar unique mapped reads and mean coverage (1,236,964,975 and 81, respectively). The coverage of exon 11 in patient's sample with the truncating R446* mutation (http://varbank.ccg.uni-koeln.de) is marked. The y-axis corresponds to the number of next generation reads covering a targeted region (exon). The x-axis corresponds to genomic coordinates. The boxes are different exons and isoforms of *PDHX*, of relevance are the blue boxes marking the protein coding exons. As mutations occur mostly in coding exons and flanking introns, these regions are targeted by the Mendeliome. Exon 1 coverage is usually lower due to high GC-content which is a challenge for targeted enrichment and sequencing of genomic regions. At least 10x reads are required to make reliable bp calls.
